# Persistent femoral neck non-union despite valgus intertrochanteric osteotomy: Relevance for secondary cam-type impingement

**DOI:** 10.1016/j.tcr.2023.100911

**Published:** 2023-08-19

**Authors:** Stefan Blümel, Matthieu Hanauer, Veerle Franken, Joseph M. Schwab, Moritz Tannast

**Affiliations:** Department of Orthopedic Surgery, HFR Cantonal Hospital, University of Fribourg, Fribourg, Switzerland

**Keywords:** Femoral neck fracture, Valgus intertrochanteric osteotomy, Femoroacetabular impingement, FAI, Secondary cam-type femoroacetabular impingement, Surgical hip dislocation

## Abstract

Valgus intertrochanteric osteotomy is a well-established treatment in delayed union of femoral neck fractures as it converts shear forces into compression forces. Non-union of the femoral neck fracture may persist following valgus intertrochanteric osteotomy, and secondary femoroacetabular impingement (FAI) may be a contributing factor.

**Case:**

We report one case of persistent femoral neck non-union after treatment by valgus intertrochanteric osteotomy with concomitant secondary cam-type impingement from fracture callus as a possible cause for ongoing insufficient healing. Healing was achieved following surgical hip dislocation with corrective osteochondroplasty of the femoral head-neck junction. Two-year follow-up shows good clinical and radiological outcomes.

**Conclusion:**

In ongoing non-healing of femoral neck fractures following valgus intertrochanteric osteotomy, secondary cam impingement from fracture callus must be excluded.

## Introduction

Valgus intertrochanteric osteotomy is still the treatment of choice for non-union of femoral neck fractures in young patients [[1], [2], [3], [4]]. Biomechanically, shear forces are converted into compression forces by repositioning the fracture line more perpendicular to the direction of force through the proximal femur [1,2,4]. Healing of the non-union can be achieved in up to 90 % of cases with good to excellent functional results in 80 % [5]. The most commonly reported reasons for failure following corrective valgus intertrochanteric osteotomy involved inappropriate surgical technique with screw cut out of the femoral head or collapse and necrosis of the femoral head [5]. More recently, secondary cam-type femoroacetabular impingement (FAI) at the fracture site [6,7] was identified as a problem in these fractures. This can be caused either by raised up cortical fragments, persisting posterior slip-like displacement of the femoral head, or prominent callus formation leading to an inclusion-type of FAI and thereby impairing healing.

We report a case of a persistent femoral neck non-union despite valgus intertrochanteric osteotomy with an initially spherical head-neck junction. In this case, the non-union was due to a reactive secondary callus formation from the compressive stress conversion at the anterior femoral head-neck junction.

## Case

**Fig. 1:**
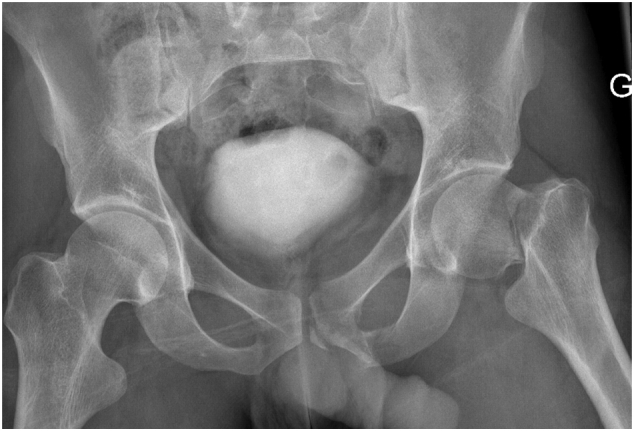
AP pelvis radiograph shows a left Pauwels type II femoral neck fracture.

A 25-year-old patient fell from a height of six meters resulting in a femoral neck fracture type Pauwels II [8]/Garden IV [9]/31B2.1 according to the AO-classification [10] (Fig. 1).

Along with the femoral neck fracture, the patient had fractures of the ipsilateral ilio- and ischiopubic ramus and along with an ipsilateral anterior compression fracture of the sacrum type Tile B 2.1 [11]/type 1 lateral-compression according to Young-Burgess [12]. These were assessed as stable, and decision was made to treat them conservatively. Closed reduction and percutaneous cannulated screw fixation of the femoral neck fracture was performed at an outside institution within 4 h of presentation using three cannulated 7.3 mm screws on a standard traction table. Post-operatively the patient was placed on 5-kg weight-bearing restriction for the operative extremity for 6 weeks.

**Fig. 2:**
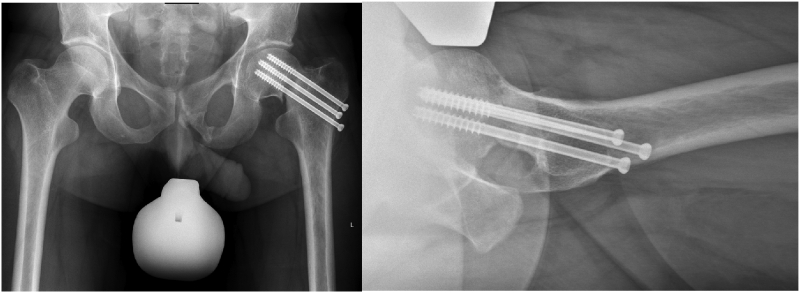
AP pelvis and cross-table lateral hip radiographs show no significant fracture union following placement of three partially threaded, 7.3 mm cannulated screws.

Six months after surgery the patient was referred to our center with a symptomatic persistent non-union of the femoral neck. On physical examination, the patient showed reduced hip mobility with pain in flexion and internal rotation. Maximum hip flexion was 90°, maximum extension was 0°, internal rotation at 90° of flexion was 10°, and external rotation at 90° of flexion was 25°. Anteroposterior (AP) pelvis radiograph and cross-table lateral radiograph of the left hip showed a femoral neck non-union, without secondary displacement, and a preserved hip joint without any signs of degeneration or femoral head collapse (Fig. 2).

**Fig. 3:**
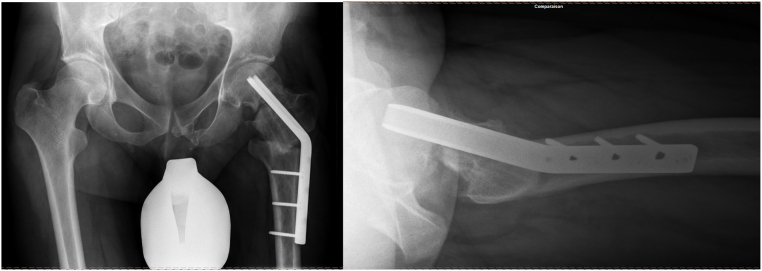
AP pelvis and cross-table lateral hip radiographs after valgus intertrochanteric osteotomy using a 130° blade plate.

Notably, the femoral head and anterior femoral head-neck junction appeared perfectly spherical on plain radiographs. After confirmation of the femoral head viability using a positron emission tomography/computed tomography (PETCT), the patient underwent valgus intertrochanteric osteotomy using a 130° blade-plate (DePuySynthes©, Zuchwil, Switzerland) 7 months after his initial injury (Fig. 3).

No offset correction was performed during the osteotomy since the femoral head-neck junction appeared spherical and showed no signs of impingement. Postoperative mobilization was started with partial weight bearing of 15 kg for 8 weeks as well as physiotherapy. Venous thromboembolism (VTE) prophylaxis was instituted with low-molecular-weight heparin. After the initial 8 weeks, weight-bearing was increased in a stepwise fashion over 6 weeks. The patient remained compliant and complained of minimal pain throughout this recovery.

At 12 months after initial trauma and 5 months after corrective valgus osteotomy, radiographs showed a healed intertrochanteric osteotomy but a persistent femoral neck non-union. In addition, at this point radiographs now showed a secondary cam-type deformity due to excessive callus formation at the femoral neck fracture site. Clinically, the patient was mobilizing without any restrictions, but reported persistent pain in daily activities and during clinical examination. ROM was slightly improved with maximum hip flexion still at 90°, maximum extension to 0°, internal rotation at 90° of flexion of 20°, and external rotation at 90° of flexion of 30°. In addition, this patient reported pain during ROM testing.

**Fig. 4:**
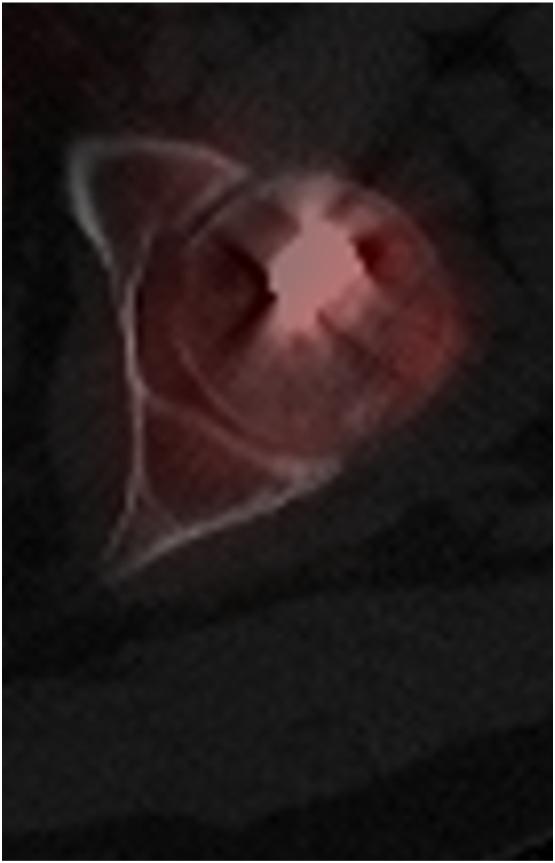
PET-CT of the left hip showing persistent femoral head vascularity.

CT imaging showed persistent non-union of the femoral neck fracture with substantial callus formation (i.e., hypertrophic pseudoarthrosis) creating a secondary cam-type deformity. In addition, CT imaging revealed a femoral antetorsion of 23°, which was elevated compared to the contralateral side [13]. The intertrochanteric osteotomy was fully healed at 7 months after revision intervention. Further work-up included hip aspiration to evaluate for intraarticular infection and repeat PET-CT to evaluate femoral head vascularity (Fig. 4).

Since the patient remained symptomatic, we discussed a surgical re-intervention to address the pain and persistent nonunion. As the hip joint remained well-preserved without signs of osteoarthritis, our recommendation was a surgical hip dislocation with osteochondroplasty of the secondary cam-deformity and revision osteosynthesis of the femoral neck non-union.

**Fig. 5:**
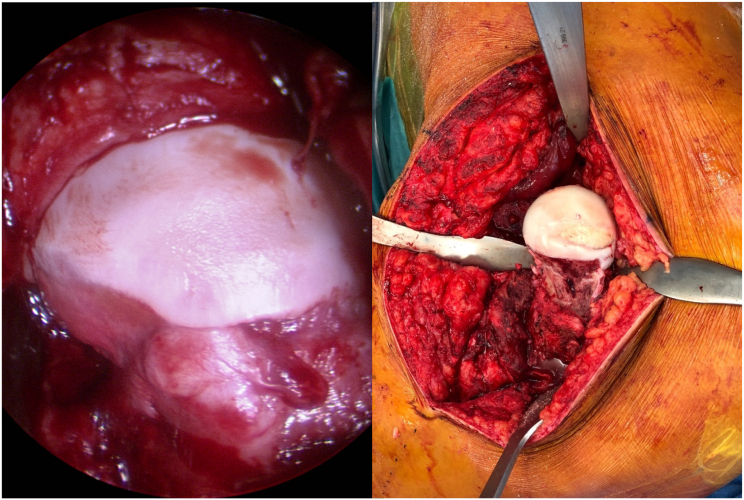
Inspection of the left hip during surgical dislocation revealed secondary callus formation on the femoral neck creating a secondary cam-type deformity.

At 20 months after initial trauma and approximately one year following valgus intertrochanteric osteotomy, we performed a surgical hip dislocation with a trochanteric step osteotomy and femoral head-neck osteochondroplasty of the hypertrophic pseudoarthrosis. Hip motion was tested intraoperatively, and this showed a clear cam-type impingement anteriorly between the hypertrophic callus and the acetabulum (Fig. 5). Inspection of the hip central compartment showed a Bern type 6 cartilage lesion with partial rupture from the 11 o'clock to 3 o'clock positions, and a depth up to 1 cm [14]. In addition, a chondro-labral separation (Bern type 5 labral lesion) from 12 o'clock to 3 o'clock was also seen [14]. The ligamentum teres showed signs of reactive inflammation (Stetzelberger ligamentous-fossa-foveolar complex grade 1) [15]. Inspection of the femoral head cartilage revealed focal degeneration at the 12 o'clock position. Femoral head necrosis, however, was formally excluded by immediate and pulsatile bleeding after drilling the femoral head with a 2.0 mm drill (Supplementary material).

**Fig. 6:**
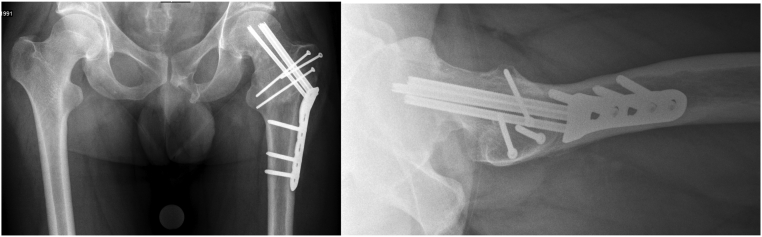
AP pelvis and cross-table lateral hip radiographs after open treatment including bone grafting, cam reduction and revision osteosynthesis with a Synthes© Pediatric hip plate.

The non-union was curetted and refreshed with multiple drillings into the fracture site. Autologous bone graft from ipsilateral greater trochanter was impacted into the non-union site to stimulate healing. After osteochondroplasty and grafting of the non-union, hip mobilization under direct visual control showed normal, impingement-free range of motion with internal rotation improved to 30° at 90° of flexion. Additionally, labral denervation was performed. Finally, revision osteosynthesis using a Synthes© Pediatric hip plate (Synthes©, Zuchwil, Switzerland) was performed to protect the femoral neck, although intraoperatively the femoral neck fracture site appeared stable (Fig. 6).

**Fig. 7:**
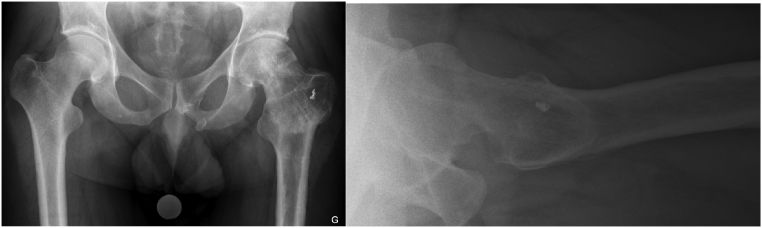
AP pelvis and cross-table lateral hip radiographs after hardware removal of the left hip.

Postoperative mobilization was started using partial weight-bearing of 15 kg for 8 weeks with limited hip flexion at 90° and no forced abduction or adduction. Follow up x-rays finally showed complete healing of the femoral neck fracture pseudoarthrosis at 6 months. At 14 months after the last intervention, hardware removal was necessary because of lateral thigh pain during physical activities (Fig. 7).

Follow up evaluation at 2.5-years after final osteosynthesis revealed a completely healed femoral neck fracture with minimal joint degeneration evident on radiographs. Clinically the patient reported a return to work with only mild intermittent hip pain with daily activities. He remained limited in heavy sporting activities. ROM was well-preserved with flexion to 90°, extension to 0°, internal rotation to 30°, and external rotation to 40°.

## Discussion

Failure of valgus intertrochanteric osteotomy for a delayed union or non-union of a femoral neck fracture is most often related to fracture malreduction, fracture comminution or vascular insufficiency [7]. We found none of these etiologies in the case presented.

Persistent non-union of the femoral neck fracture due to secondary cam-type FAI is a newly-described and rare etiology caused by hypertrophic femoral neck pseudoarthrosis [7]. This situation requires a broad approach with revision osteosynthesis in the correct anatomic position, and concomitant offset correction to remove the impingement. Without addressing both issues, symptomatic coxarthrosis through either femoral head necrosis or FAI-related joint degeneration may occur [7]. While callus formation can produce relative fracture stability resulting in potentially limited initial clinical symptoms, increased activity may lead to increased symptoms related to FAI.

As a separate pathology, CAM-type FAI seen after malunited femoral neck fractures has been described [16]. Typically, these patients present with groin pain and a positive anterior impingement test [17]. When FAI is the result of a malunion, such as in femoral neck retrotorsion or varus, these can often be treated with femoral offset correction [6,18]. In major deformities, revision surgery with corrective intertrochanteric flexion-valgus osteotomy, such as those used in correction of late-slipped capital femoral epiphysis, might be useful [19].

We routinely perform concomitant offset correction during primary intervention when impingement is visible under fluoroscopy or a clear conflict exists during ROM assessment. We often perform this offset correction through an additional anterior arthrotomy as it allows visual control of the impingement.

In older patients or patients with other limiting factors, partial or total hip replacement (THA) may be initially indicated as it allows fast recovery and early mobilization and a possible reduction in subsequent interventions [20]. There is still no clear consensus regarding the exact age limit after which arthroplasty should be performed [20].

However, in younger patients with well-preserved articular cartilage and hip joint function, we aim to conserve the native joint. Femoral head perfusion needs to be assessed, either radiographically with scintigraphy, or intraoperatively drilling the femoral head [[21], [22], [23]] prior to deciding on the final surgical treatment. This is especially indicated in the setting of revision for pseudoarthrosis. To obtain sufficient bone stock and allow stable healing, decortication and bone grafting from the greater trochanteric region or the iliac [24,25] may be performed. Ultimate stabilization in anatomic position should be achieved using constructs that prevent secondary displacement.

In addition, a torn or degenerated labrum can present an additional pain generator. This can be treated with labrum denervation for pain relief [26]. Until today no chondral grafting intervention has objectively been able to show persisting cartilage restoration in relevant chondral damage of the hip joint. To limit cartilage degeneration at the time of re-intervention, diagnosis and intervention should be performed as early as possible.

Careful clinical and radiographic evaluation is necessary to assess fracture pattern, fracture reduction, and joint degeneration to decide on an ideal intervention.

## Conclusion

Secondary CAM impingement caused by hypertrophic callus formation in persistent femoral neck fracture non-union, even after appropriate treatment by valgus osteotomy, must be excluded. To obtain complete healing and good clinical results in this scenario, offset correction, bone grafting, and revision fixation through surgical hip dislocation should be considered.

## Statement of informed consent

Consent was obtained from the patient. No ethical approval was necessary due to the use of information already obtained during previous regular follow-up controls from completely anonymized patient data without any risk of harming the patients through the use of the data.

## Declaration of competing interest

The authors declare that they have no competing interests. This research received no specific grant from any funding agency in the public, commercial, or not-for-profit sectors.
